# Improvement of the Safety Profile of Canaloplasty and Phacocanaloplasty: A Review of Complications and Their Management

**DOI:** 10.1155/2020/8352827

**Published:** 2020-06-16

**Authors:** Joanna Konopińska, Zofia Mariak, Marek Rękas

**Affiliations:** ^1^Department of Ophthalmology, Medical University of Białystok, M. Sklodowska-Curie 24A STR, Białystok 15-276, Poland; ^2^Department of Ophthalmology, Military Institute of Medicine, Szaserów 128 STR, Warsaw 04-141, Poland

## Abstract

Research on the methods used to achieve persistent and safe control of intraocular pressure resulted in the implementation of novel surgical procedures, such as canaloplasty and phacocanaloplasty. Herein, we review the literature focused on the safety profile of canaloplasty and phacocanaloplasty and the management of related complications. The aim of canaloplasty is to restore the natural aqueous outflow. This goal is achieved via a surgical procedure that involves viscocanalostomy with catheterisation of Schlemm's canal (360°) and placement of a circumferential suture that tensions the canal walls. This improves Schlemm's canal drainage, choroidoscleral flow, and subconjunctival filtration. The efficacy of canaloplasty for reducing the intraocular pressure is similar to those of trabeculectomy with mitomycin C and deep sclerectomy augmented with an implant and mitomycin C. However, canaloplasty is associated with a lower complication rate than those conventional techniques. Novel microsurgical techniques for the treatment of glaucoma are unlikely to replace the conventional methods. However, these new techniques offer alternatives, especially for patients who have an early indication for surgical intervention. Nevertheless, canaloplasty is associated with the expectations of efficient, safe, and modern surgical treatment.

## 1. Introduction

The conventional hypotensive treatment of glaucoma is based primarily on pharmacotherapy, whereas surgery is a secondary option. However, the lower postoperative complications rates resulting from improved surgical techniques justify a reconsideration of this approach. Surgical treatment provides more efficient control of the intraocular pressure (IOP) and often eliminates the patient's chronic use of eye drops. While the actions of pharmaceutical agents are limited to inhibiting the production of aqueous humour or improving its outflow via natural routes, the aim of surgical treatment is to improve the aqueous outflow by facilitating its drainage through Schlemm's canal (deep sclerectomy), by forming a new, alternative route of outflow within the trabecular meshwork (trabeculectomy) or by evacuating fluid via artificial filtering fistulae (seton surgeries). Research on the methods for persistent and safe control of IOP resulted in the implementation of novel surgical procedures such as canaloplasty and phacocanaloplasty. An awareness of the possible complications and the ability to cope with these events may significantly improve the postoperative results. Herein, we review the literature focused on the safety profiles of canaloplasty and phacocanaloplasty and the management of related complications.

## 2. Main Text

### 2.1. Overview of Canaloplasty

Canaloplasty has been used for nearly 15 years [1]. The iTrack microcatheter (Ellex Medical Ltd, Adelaide, SA, Australia) was first introduced to the glaucoma surgical armamentarium in 2005 in the context of the viscocanalostomy procedure, in which Schlemm's canal undergoes viscodilation [2, 3]. This procedure was first described by Stegmann as a nonpenetrating, filtering, and bleb-independent version of Schlemm's canal dilation. This technique has evolved with the addition of an intracanalicular tension suture to the canaloplasty procedure.

**Figure 1:**
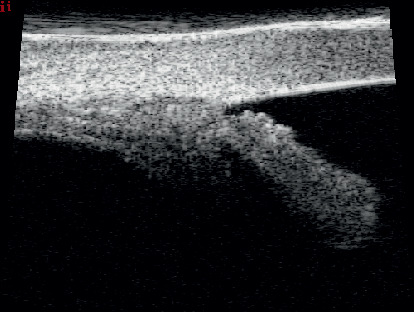
Collapsed Schlemm's canal before canaloplasty.

**Figure 2:**
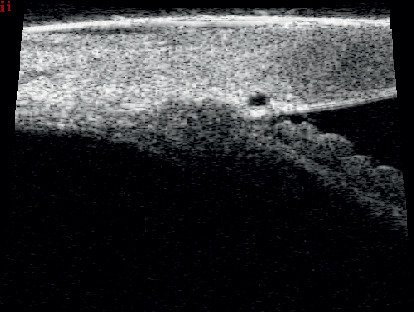
Dilation of Schlemm's canal is visible in ultrasound biomicroscopy (UBM).

Data from the literature suggest that this technique may constitute an alternative to the conventional surgical treatment of open-angle glaucoma. The tensioning of the canal wall, especially the trabecular meshwork, and the restoration of physiological aqueous outflow are reflected by a marked hypotensive effect [3]. This mechanism can be compared to the effect of pilocarpine on the trabecular meshwork [4]. During viscodilation, the profile of Schlemm's canal changes from an oval to a round shape, which can be visualized clearly using an 80 Hz ultrasound probe (Figures 1 and 2) [5]. However, the procedure is not entirely free of limitations, as it does not provide a persistent decrease in IOP below the blood pressure in the episcleral veins.

In selected cases of concomitant glaucoma and cataract, the glaucoma surgery can be combined with phacoemulsification, which may enhance the positive effect of the former on the IOP. The hypotensive effect of the combined surgery was shown to be 2 mmHg better than that of canaloplasty alone [3, 6]. Lewis et al. [4] defined a complete therapeutic success as an IOP ≤ 18 mmHg without the use of hypotensive medication. This milestone was achieved in 36% of individuals after canaloplasty and in up to 70.4% of patients subjected to the combined procedure. Moreover, fewer patients subjected to phacocanaloplasty require an adjunct goniopuncture to achieve the target IOP, compared to those who undergo canaloplasty alone [6]. The efficacy of these procedures is also reflected by the 53% and 80% reductions in the use of hypotensive eye drops after canaloplasty and combined surgery, respectively [3]. However, an opposite effect may be observed if phacoemulsification is performed together with conventional trabeculectomy, which stimulates a stronger inflammatory reaction [7, 8].

The principal advantage of canaloplasty over conventional trabeculectomy is that the former procedure is associated with fewer complications that are usually associated with the presence of a filtering bleb [1, 2] or corneal complications [9]. Tight closure of the superficial scleral flap eliminates problems such as patient discomfort, wound leakage, and intraocular infections [10]. However, the lower morbidity rates after canaloplasty do not exempt ophthalmic surgeons from understanding the potential risks inherent to this procedure and the associated management.

### 2.2. Complications Associated with Canaloplasty and Phacocanaloplasty

**Figure 3:**
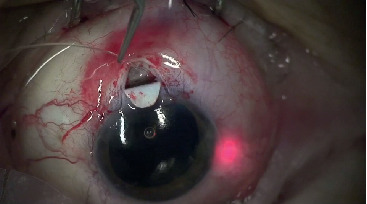
The incorrect passage of the catheter within the canal.

The most prevalent intraoperative complications of canaloplasty include the inability to insert a microcatheter into Schlemm's canal and the incorrect passage of the catheter within the canal [4, 10, 11]. The reported rates of successful cannulation of the canal range from 74% [4] to 89.9% [10]. Problems with intubation may occur in cases involving unexpected structural anomalies of Schlemm's canal or in patients with a history of laser surgeries involving the filtration angle (argon laser trabeculoplasty) that resulted in the destruction of the trabecular meshwork and excess cicatrisation [12] (Figure 3).

According to Grieshaber et al. [13], a microcatheter may disrupt the wall of Schlemm's canal and penetrate the anterior chamber or suprachoroidal space in 3.3% of cases. Although this event may cause the leakage of viscoelastic material to the anterior chamber and a transient postoperative increase in the IOP in the former case, it may cause limited ciliary body detachment that could potentially lead to hypotony in the latter. Lewis et al. [4, 10] reported a case in which Schlemm's canal was cut, and a suture was extruded through to the anterior chamber. Such a situation may result from the excessive tension of a thread during placement of the suture or the inappropriate removal of a microcatheter during insertion of a thread. The vector of the force placed on a microcatheter during the retrograde insertion of a suture should be curved in parallel with the corneal curvature. In approximately 10% of the cases, in which catheterisation of Schlemm's canal is impossible, and an operator may perform a deep sclerectomy or conversion to trabeculectomy [7, 8].

**Figure 4:**
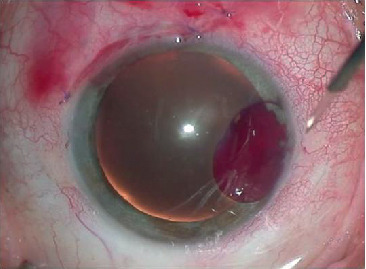
Haemorrhagic detachment of Descemet's membrane.

Descemet's membrane detachment is a less frequent but important intraoperative complication, as it is reported in 1.6–9.1% of cases and potentially leads to a loss of vision (Figure 4) [4, 10, 14–16].

Usually, detachment occurs in the inferior temporal and inferior nasal quadrants or less commonly in the superior temporal quadrants [16], and it typically arises during the passage of a microcatheter through Schlemm's canal. The direct causes of this complication remain unknown. The aetiology of the complication is explained by two hypotheses [16]. According to the first hypothesis, the viscoelastic material may reach a critical mass in the inferior quadrant when the microcatheter is administered superiorly in the 12 o'clock position. The resistance of the canal during the passage of a microcatheter and the stretching of the canal walls by a viscoelastic material may exceed the durability of the termination of Descemet's membrane at Schwalbe's line, leading to detachment. According to the second hypothesis, either the canal may be less resistant in the inferior quadrants or some patients may be genetically predisposed to this complication; the latter appears to be supported by the fact that certain individuals are present with bilateral Descemet's membrane detachment [17]. This complication is postulated to be more frequent in eyes with adhesions between the outer and inner walls of the canal [16]. Additionally, the role of the operator and an imperfect surgical technique cannot be excluded as causes. The too-slow and noncontinuous passage of a microcatheter through the canal and the simultaneous excessive administration of viscoelastic material can cause a rupture of the canal wall at the termination of Descemet's membrane [17]. Although the detachments are generally small (1-2 mm), they may reach up to 5-6 mm in size and extend into the visual axis. Sometimes, the area below the detachment is filled with the viscoelastic material or blood, which is classified as a haemorrhagic detachment of Descemet's membrane. Bleeding is a consequence of blood reflux from the episcleral veins to the previously collapsed canal, which occurs in cases wherein the pressure in the anterior chamber decreases below that in the episcleral veins [18]. This complication may manifest intraoperatively or during the initial 24-hour postoperative period [19]. Inferior quadrants contain a greater number of collector channels that communicate with episcleral veins, which promote the development of reflux and bleeding in the inferior hemisphere [20].

The management of Descemet's membrane detachment depends on the size and the resorption rate of the substance filling the area below the detachment. Expectant management is recommended in the case of a small detachments filled with a viscoelastic material or small volume of blood, as these usually spontaneously resorb within approximately 1–6 weeks. Sometimes, a persistent brownish clouding of the cornea caused by the residues of haemolysed blood may be observed for up to 3 months postoperatively [21]. Large detachments involving the visual axis, especially those filled with blood mixed with viscoelastic material, should be evacuated because these can cause impaired visual acuity and a secondary enhanced loss of the corneal endothelium; the latter, if left untreated for 3 months, may lead to persistent corneal decompensation [21] and would constitute an indication for penetrating keratoplasty [16].

Several treatment options can be implemented in a case of this complication. External drainage can be performed postoperatively by puncturing the external layers of the cornea and simultaneously administering air, viscoelastic material, or sulphur hexafluoride into the anterior chamber to enforce the spontaneous evacuation of blood [16]. Some authors prefer to perforate Descemet's membrane *ab interno* and evacuate the haematoma from the anterior chamber [22]. Others recommend the active lavage of fluid and blood from under the detachment with the aid of a Rycroft cannula [23]. Another less invasive management option involves the evacuation of the contents under the detachment from the anterior chamber using an Nd : YAG laser [18]. The laser beam should be targeted on a point located at +1.25 posteriorly to the endothelial surface, without using a contact lens and with the power set at 1.2 mJ per impulse. Two impulses are then used to make two small perforations in the lower part of Descemet's membrane adjacent to the area of detachment, which enables the evacuation of the liquefied blood from the anterior chamber. The presence of blood is associated with an increased risk for vision-endangering complications such as corneal oedema, corneal staining, endothelial defects, ulceration, corneal thinning, posterior keratoconus formation, and close-angle glaucoma developing secondary to a pupillary block or resulting from clot translocation to the anterior chamber [20]. Some authors recommend the immediate evacuation of a haematoma during primary surgery via a corneal incision at the two ends of the detachment area and the mechanical evacuation of clots and fluid with the aid of a spatula and Osher forceps [19]. After evacuating most of the contents, the larger corneal incision should be closed with 10/0 sutures, whereas the smaller incision should be left open. Simultaneously, air should be pumped into the anterior chamber to enforce the evacuation of the remaining fluid with the patient's movements during the postoperative period.

**Figure 5:**
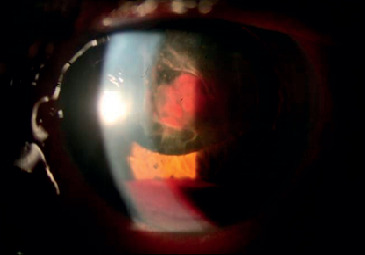
Hyphaema, corneal oedema, and anterior chamber inflammation.

Complications are usually observed during the early phase (within the initial 90 days postoperatively) and exhibit a marked decrease in incidence at >90 days postoperatively [6]. The most typical postoperative complication is the collection of blood in the anterior chamber (Figure 5). This complication is observed in 6.1–85.2% of cases involving all single and combined procedures [3, 10,11, 15, 24]. This complication is normally transient and resolves spontaneously within 7–28 days. Hyphaema (microhyphaema) is defined as a small haemorrhage in the anterior chamber that is composed of liquid blood without clots. The approximate height of a hyphaema is usually 1–2.5 mm. Hyphaemas result from hypotony in the anterior chamber and inherent blood reflux from the episcleral veins to Schlemm's canal and via the eye's porous trabecular meshwork to the anterior chamber. The presence of hyphaema indicates an appropriate tensioning of the canal walls by an inserted thread and the restoration of a physiological outflow system. Therefore, hyphaema may be considered an indirect marker of successful canaloplasty, especially when the appropriate tensioning of a thread during the placement of the suture cannot be determined using any other objective method. Consequently, the absence of a hyphaema can be interpreted as the insufficient tensioning of a suture inside the canal [9]. This hypothesis is supported by the results of previous studies, in which complete surgical success (IOP < 16 mmHg) was achieved in 87% of patients with a hyphaema and in only 21% of individuals without hyphaema [9]. Moreover, patients in the latter group more often required additional postoperative goniopuncture to achieve the target IOP. Paradoxically, larger hyphaemas may be resorbed faster than smaller ones because the former is associated with higher permeability in the trabecular meshwork and better patency in the collector channel system [9]. This situation differs from that of haemorrhages formed during or after trabeculectomy, which occurs at an estimated incidence of 3–43% [7, 9, 24]. Haemorrhages result from an injury to the trabecular meshwork or the iris, as well as a reflux of blood from the conjunctival vessels, and may impair vision. Consequently, the bleeding may be more severe and persistent, may tend to recur, and can cause permanent corneal imbibition [7, 9].

Although corneal oedema occurs slightly more frequently after phacocanaloplasty than after canaloplasty, it resolves within 7 days without persistent visual impairment, irrespective of the type of surgery [6]. Inflammation of the anterior chamber, which manifests as a clouding of the chamber fluid, occurs more frequently after combined surgeries. This effect is observed within 24 hours postoperatively and usually resolves within 7 days following the administration of topical corticosteroids.

Some patients (1.6–18.2%) may exhibit a transient increase in the IOP of up to >30 mmHg, probably due to the presence of viscoelastic material in the anterior chamber after phacocanaloplasty or viscoelastic penetration through the wall of Schlemm's canal to the chamber lumen after canaloplasty [12]. In 8.3–18.8% of cases and particularly in those involving single procedures, the increase in IOP may persist for longer. Laser goniopuncture is an efficient treatment option in such cases. This adjunct treatment is required in 4.7–27.3% of cases. An Nd : YAG laser goniopuncture should include 15 impulses of 2–4 J each, which are targeted on the trabeculodescement membrane. A small fraction of patients may require repeated goniopuncture [3], and a trabeculectomy or a seton procedure may be considered in such cases.

Some studies involving a long-term follow-up period documented the formation of cataracts after canaloplasty in phakic eyes, although the data on this topic are inconclusive [3, 12]. The incidence of this complication is estimated to range from 12.7% to 19.1% [3, 10] and is lower than the incidence after trabeculectomy (24–47%). According to Wang et al., the mean elapsed time between canaloplasty and phacoemulsification in patients with significant progression of cataract is 24 months. Although phacoemulsification impairs the hypotensive effect of a successful trabeculectomy [25], canaloplasty does not result in a long-term increase in the pressure but rather causes a further slight reduction in the pressure [6].

**Figure 6:**
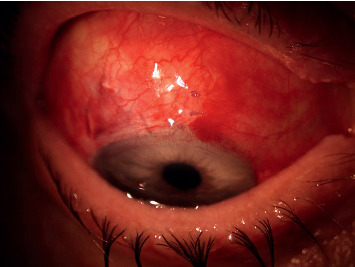
Filtering bleb after canaloplasty.

Although canaloplasty is by default a filtering bleb-independent procedure, the presence of the latter is reported in 2.5–10% of cases, especially after the performance of combined surgeries (Figure 6) [3, 6].

To date, no cases of endophthalmitis attributable to canaloplasty have been reported. In contrast, the incidence of endophthalmitis after trabeculectomy reached up to 9.6% and increases by 2.5% per patient-year with the addition of mitomycin C [12]. Hypotony is a very rare complication of canaloplasty observed in 0.6% of cases. In contrast, trabeculectomy-related hypotony is markedly more frequent, affecting between 13.8% [26] and 43% of patients [27]. Other rarely reported complications of canaloplasty include a late extrusion of the intracanal suture through the trabecular meshwork to the anterior chamber, the extrusion of sutures closing the scleral flap, choroidal exudate, bleeding from the wound, and anterior adhesions at the area of the trabeculodescement window [8, 9]. Severe complications, such as choroidal detachment, suprachoroidal haemorrhage, inflammation of a filtering bleb, endophthalmitis, a shallow anterior chamber, or malignant glaucoma, have not been reported in patients subjected to canaloplasty.

## 3. Conclusions

Novel microsurgical techniques for the treatment of glaucoma are unlikely to replace the conventional methods. Rather, these techniques offer new alternatives, especially for patients who have an early indication for surgical intervention. Canaloplasty is a relatively new procedure, and its long-term (i.e., 20–30 years) effectiveness and complications remain unknown. Accordingly, we think that this procedure should be the focus of further research. This review does not address the modifications of the standard canaloplasty, such as minicanaloplasty or canaloplasty *ab interno*, as these topics are the subject of another paper by the authors [2]. Nevertheless, canaloplasty is associated with the expectations of an efficient, safe, and modern surgical treatment.

## Data Availability

All data generated or analysed during this study are included within the article.
